# Unilateral renal agenesis, blind-ended ureter, and ectopic ureterocele inserting into the seminal vesicle: A very rare developmental association

**DOI:** 10.1016/j.eucr.2023.102505

**Published:** 2023-07-13

**Authors:** Sara Sorour, Craig Ferguson, Mitchell P. Wilson, Gavin Low

**Affiliations:** University of Alberta Department of Radiology and Diagnostic Imaging, 2J2.00 WC Mackenzie Health Sciences Centre, Edmonton, Alberta, Canada

**Keywords:** Renal agenesis, Blind-ended ureter, Ectopic ureterocele

## Abstract

Congenital renal anomalies are common imaging findings and can often be detected antenatally. In some cases, these anomalies may go undetected and present in adulthood. We report a very rare case of unilateral renal agenesis in a 22-year-old male associated with an ipsilateral dilated blind-ended ureter that ectopically inserted into the seminal vesicle. This unique combination of developmental anomalies can lead to a variety of clinical presentations and requires careful monitoring and management.

## 1 Introduction

Unilateral renal agenesis is a congenital condition where one kidney is absent due to incomplete development during embryonic growth. Most patients with unilateral agenesis remain symptom free as the contralateral kidney can compensate with adequate renal function. To the best of our knowledge, less than 10 studies in the medical literature have reported the combined occurrence of unilateral renal agenesis, blind-ending ureter, and ureterocele. However, the addition of an ectopic ureterocele inserting into the seminal vesicle has not been previously reported.

## 2 Case presentation

**Fig. 1:**
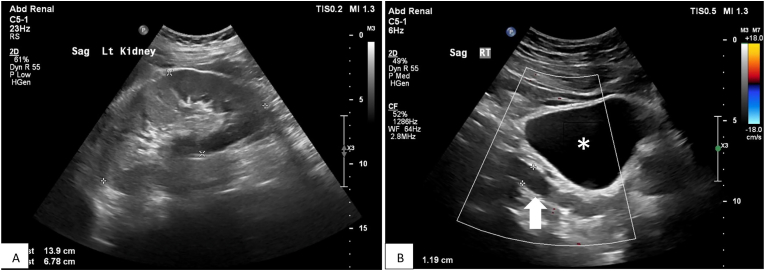
(A) ultrasound confirms the known finding of an absent right kidney and shows a hypertrophic left kidney that measured 13.9 cm in length. (B) ultrasound shows a tubular anechoic fluid filled structure (arrow) posterior lateral to the right side of the bladder (*).

**Fig. 2:**
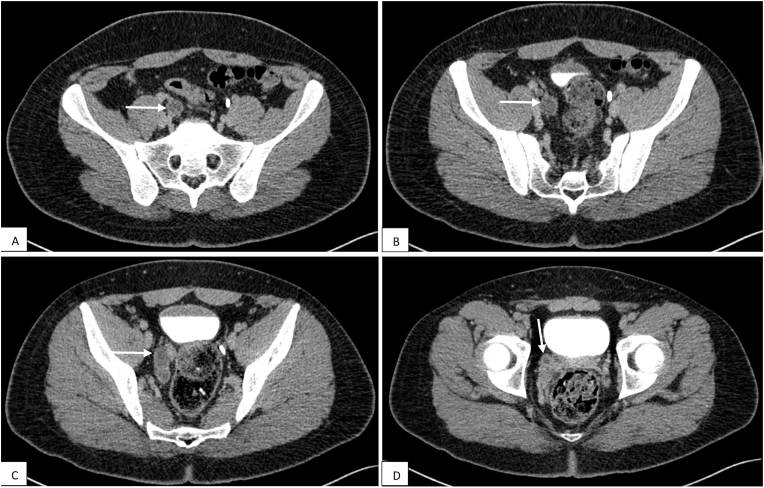
(A–C) Axial CT urogram at 8 minute post intravenous contrast injection shows a blind-ended dilated right ureter (arrows in A-C). The distal ureter inserts ectopically into the right seminal vesicle (arrow in D).

**Fig. 3:**
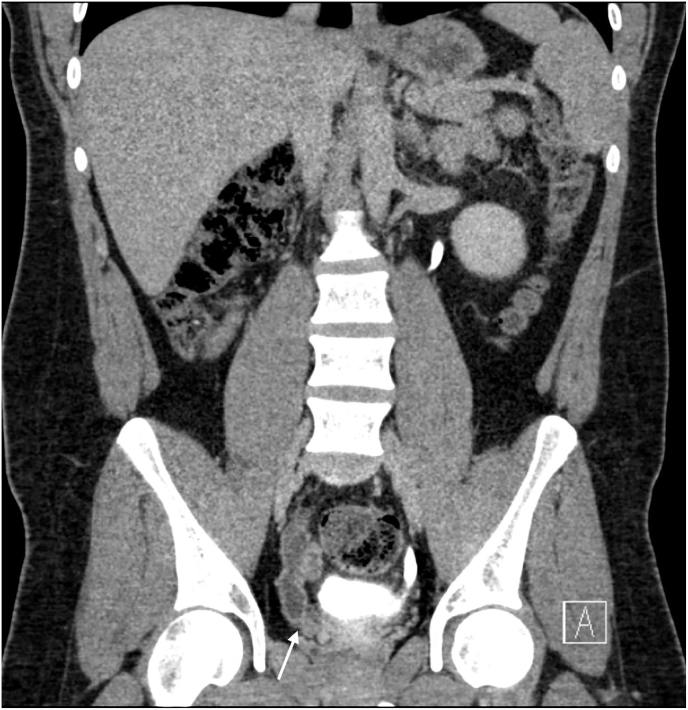
Coronal reconstructed CT urogram of the abdomen and pelvis shows a blind-ended dilated right ureter that inserts ectopically into right seminal vesicle (arrow).

A 22-year-old male with known Kallmann syndrome presented with penile pain for 3 months with an episode of gross hematuria, and no symptoms of urinary tract infection. The patient was a non-smoker with a no personal or family history of renal or urologic cancer. Relevant investigations including urinalysis, urine culture and urine cytology were normal. Ultrasound showed right renal agenesis (previously diagnosed), a normal left kidney and bladder, and an incidentally noted 7 cm vertically orientated dilated tubular fluid filled structure in the right retroperitoneum that passed posterolateral to the bladder and inserted into the right seminal vesicle. The differential diagnosis included a residual ureter or an enlarged seminal vesicle (Fig. 1). Cystoscopy was performed showing a normal left ureteric orifice but a right ureteric orifice was not identified. No other abnormality was visualized on cystoscopy to explain the penile pain or the lower urinary tract symptoms. A CT urogram confirmed a remnant dilated right ureter with a blind-ending proximal portion, and a distal ectopic insertion into the right seminal vesicle (Fig. 2, Fig. 3).

## 3 Discussion

Renal agenesis is a congenital anomaly with an incidence of approximately 5 in 10,000 live births, more frequently occurs on the left, and has a male predilection. In our case, the patient is known to have right renal agenesis associated with Kallmann Syndrome. Kallmann Syndrome entails hypogonadotropic hypogonadism with anosmia or hyposmia, midline defects, cleft lip and palate, renal agenesis, and neurosensory deafness. The prevalence has been estimated at 1 in 10,000 males and 1 in 50,000 females.^1^

The ureters normally implant in the urinary bladder at the superolateral angle of the trigone. Ectopic ureter refers to a ureter that inserts outside its normal anatomical location. Ureterocele is an abnormal dilatation of the distal ureter, which can be either intravesical or ectopic. The combination of renal agenesis, a proximal blind-ended ureter, ureterocele, and ectopic insertion into the seminal vesicle is extremely rare, and to our knowledge, has not been previously reported in the English-speaking literature.

The clinical presentation in patients with a combination of renal agenesis, blind-ended ureter and ureterocele varies widely. For example, in our case the patient presented with gross hematuria and penile pain, and no urinary tract infection symptoms. In Saadeh et al.^2^, the patient was asymptomatic, and the abnormality was incidentally found on CT performed for a different clinical question. In Ahmed et al.^3^, the patient presented with lumbar pain, recurrent urinary tract infection but no fever. In Mohseni et al.^4^, the patient presented with unilateral lower quadrant pain and fever without rebound tenderness or guarding. In Maas et al.^5^, one of the patients presented with intermittent symptoms of urinary tract obstruction and left groin pain with probable unilateral pelvic mass, which by initial ultrasound correlated to an ipsilateral seminal vesicle cyst. The second patient, known to have dysplastic unilateral kidney, presented with recurrent epididymitis and worsening urinary frequency.^5^

Complications of unilateral renal agenesis includes high blood pressure, proteinuria, and chronic kidney disease that may progress to end-stage ultimately requiring dialysis or a kidney transplant. Therefore, it is necessary to investigate congenital urinary tract anomalies early to avoid delay in diagnosis or complications associated with these anomalies which could result in chronically impaired renal function. Providing that unilateral renal agenesis with complications is confirmed by imaging, surgery is the definitive option to excise the blind-ended ureter in order to save the contralateral urinary tract from reflux, ascending infection, and related complications.^3^ In our case, the patient once symptoms resided and subsequently, he was treated conservatively with urology follow-up.

## 4 Conclusion

We present a unique case involving a combination of developmental urologic anomalies: renal agenesis, blind-ended ureter, ureterocele, and ectopic ureteric insertion into the seminal vesicle. The clinical presentation can be asymptomatic or present with symptoms such as pain, hematuria, and symptoms of a urinary tract infection.

## Declaration of patient consent

Patient's consent not required as patient's identity is not disclosed or compromised.

## Financial support and sponsorship

Nil.

## Declaration of competing interest

There are no conflicts of interest.
